# Effects of Cyclodextrins (β and γ) and l-Arginine on Stability and Functional Properties of Mucoadhesive Buccal Films Loaded with Omeprazole for Pediatric Patients

**DOI:** 10.3390/polym10020157

**Published:** 2018-02-07

**Authors:** Sajjad Khan, Joshua Boateng

**Affiliations:** Department of Pharmaceutical, Chemical and Environmental Sciences, Faculty of Engineering and Science, University of Greenwich at Medway, Central Avenue, Chatham Maritime, Kent ME4 4TB, UK; sajjadkhan_1@hotmail.com

**Keywords:** buccal mucosa drug delivery, cyclodextrins, films, l-arginine, mucoadhesive polymer, omeprazole, paediatric

## Abstract

Omeprazole (OME) is employed for treating ulcer in children, but is unstable and exhibits first pass metabolism via the oral route. This study aimed to stabilize OME within mucoadhesive metolose (MET) films by combining cyclodextrins (CD) and l-arginine (l-arg) as stabilizing excipients and functionally characterizing for potential delivery via the buccal mucosa of paediatric patients. Polymeric solutions at a concentration of 1% *w*/*w* were obtained by dispersing the required weight of metolose in 20% *v*/*v* ethanol as solvent at a temperature of 40 °C using polyethylene glycol (PEG 400) (0.5% *w*/*w*) as plasticizer. The films were obtained by drying the resulting polymer solutions at in an oven at 40 °C. Textural (tensile and mucoadhesion) properties, physical form (differential scanning calorimetry (DSC), X-ray diffraction (XRD) and Fourier transform infrared (FTIR) spectroscopy), residual moisture content (thermogravimetric analysis (TGA)) and surface morphology (scanning electron microscopy (SEM)) were investigated. Optimized formulations containing OME, CDs (β or γ) and l-arg (1:1:1) were selected to investigate the stabilization of the drug. The DSC, XRD, and FTIR showed possible molecular dispersion of OME in metolose film matrix. Plasticized MET films containing OME:βCD:l-arg 1:1:1 were optimum in terms of transparency and ease of handling and therefore further functionally characterized (hydration, mucoadhesion, in vitro drug dissolution and long term stability studies). The optimized formulation showed sustained drug release that was modelled by Korsmeyer–Peppas equation, while the OME showed stability under ambient temperature conditions for 28 days. The optimized OME loaded MET films stabilized with βCD and l-arg have potential for use as paediatric mucoadhesive buccal delivery system, which avoids degradation in the stomach acid as well as first pass metabolism in the liver.

## 1. Introduction

Gastro-oesophageal reflux involves movement of excessive acid from the stomach into the oesophagus. It affects a significant number of infants, exhibiting an array of symptoms, including physiological reflux, hematemesis, or even sudden infant death syndrome. Many of these children suffer from gastro-oesophageal reflux but with no definite causes or accompanying complications [1]. In addition, some investigations that assessed the effects of early therapeutic interventions showed that about 55% of the infants are free of any clinical symptoms by the time they are 10 months old and this increases to 81% by 18 months old [2]. That notwithstanding, it is still vital to identify which children exhibit gastro-oesophageal reflux-associated disease to allow selection of the most effective therapy for treating the manifestations of the disease [3].

Until recently, Cisapride which possesses 5HT-4 antagonist characteristics, was the drug of choice in gastro-oesophageal reflux [4]. However, due to cardiac dysrhythmias associated with its use, Cisapride is no more routinely prescribed for the condition. Although histamine receptor 2 antagonists (H_2_RAs) can provide relief from oesophagitis, and high-dosage ranitidine (20 mg·kg^−1^·day^−1^) demonstrated as effective in refractory reflux oesophagitis, side effects such as nocturnal acid secretion present a limitation [5]. It has been shown that pharmacological tolerance of proton pumps inhibitors (PPIs) is good, safe for patients and an effective way of treating gastro-oesophageal reflux disease (GERD) [6]. PPIs are chemical compounds that inhibit hydrogen/potassium adenosine triphosphatase enzymes in the stomach wall, and providing relief from oesophageal, gastric and duodenal ulcers as well as from GERD [5]. They act by changing their chemical structure upon interaction with H^+^/K^+^ adenosine triphosphatase enzyme in the parietal cells, which leads to the formation of its active derivative through acceptance of a H^+^ proton, thereby increasing stomach pH whilst reducing the secretion of acid from the stomach wall. Furthermore, the protonated derivative can bind with parietal cells in the stomach wall, and subsequently lowers the secretion of acid even further [7]. PPIs can be divided into: (i) competitive; and (ii) covalent, with the competitive PPIs exerting reversible inhibition of the proton pump mechanism in the wall of the stomach by binding to its extracellular surface. On the other hand, PPIs belonging to the covalent class cause inhibition, which is not reversible, and results in a longer period for the secretion of other enzymes. Initially, 2-methyl-8-(phenyl-methoxy)-imidazo-1,2-pyridin-3-acetonitrile and 3-butyryl-8-methoxy-4-(2-tolylamino) quinolone were the most common PPIs, however, more recent groups are composed mainly of benzimidazole derivatives such as omeprazole and pantoprazole [8].

Omeprazole (OME) is an effective therapy for treating ulcers of the stomach and duodenum often combined with antibacterial drugs to eliminate *Helicobacter pylori* [9]. For children suffering from GERD and demonstrating acute symptoms as well as those with erosive, ulcerative, or stricturing (narrowing) of the oesophagus caused by using endoscopy, a starting course of OME is the therapeutic regimen of choice [10]. OME is also used to prevent and treat ulcers resulting from using non-steroidal anti-inflammatory drugs (NSAID), and the dose of OME should normally be maintained in such situations even after healing of the ulcer to avoid chances of recurring [11]. Furthermore, OME is effective for treating Zollinger–Ellison syndrome and also employed to aid in reducing degradation of pancreatic enzyme supplements in children suffering from cystic fibrosis [8]. 

However, although OME is effectively absorbed from the gastrointestinal tract, the systemic bioavailability after oral administration is between 40% and 50% which suggests that the drug experiences significant first pass metabolism in the liver. Once it is absorbed, OME gets metabolized into three main metabolites: OME sulphone, OME sulphide and hydroxyl OME, all of which have been detected in human plasma [12]. Hydroxylation position 5 is subject to genetic polymorphism and the sulphone in plasma is accumulated in patients who metabolize S-mephenytion 4′ hydroxylation poorly [13]. Another challenge with the drug is that, in aqueous solution, OME’s stability is solely determined by the pH and rapidly degrades under acidic and neutral conditions, but shows better stability in alkaline environments [14]. OME is also rapidly degraded by heat, light and humidity [15]. These limitations present a formulation challenge in the design and manufacture of oral pharmaceutical delivery systems with optimum bioavailability due to its rapid gastric degradation [9]. To avoid such stomach acid breakdown, OME is formulated as enteric-coated granules in the form of capsules [2]. As a result, alternative formulations for administration via non-enteric routes such as buccal mucosa have been proposed [16,17]. These notwithstanding, the physical instability of OME remains an issue during formulation and storage and therefore requires stabilizing agents such as l-arginine and cyclodextrins.

Cyclodextrins (CDs) are oligosaccharides with cyclic configurations employed as excipients in different fields such as the preparation of inclusion complexes utilized in various dosage forms. They are able to form water-soluble complexes with poorly water soluble drugs which fit into their cavities [18]. The three main types of CDs are α, β and γ comprising 6, 7 and 8 d-glucose units respectively. The molecular structure of CDs involves glucopyranose units in 4C1-chair conformation connected through α (1 → 4) bonds. The glucose units are syn-oriented in which O-6 hydroxyls are on one side of the ring while the O-2 and O-3 hydroxyls are on the other side. The internal hydrophobic cavity of CDs facilitates their formation of inclusive complexes which allows their effective use as a drug carrier to improve drug solubility, chemical stability, dissolution and bioavailability or to decrease unfavourable side effects.

l-arginine (*S*-2-amino-5-guanidinopentanoic acid) is an amino acid which is basic in nature and presents naturally in human diets, especially foods such as meats and nuts. Arginine prevents the involution of thymic after surgery and helping in the increase of lymphocytes which is an essential agent for adequate wound healing. In addition, arginine is used as an auxiliary substance to increase the water molecular solubility of other compounds [19].

Metolose (MET) is a non-ionic water-soluble cellulose ether that is derived from pulp. Highly purified pulp is etherified with methyl chloride or a combination of methyl chloride and propylene oxide to form a water soluble, non-ionic cellulose ether. It can produce transparent films by casting from their gel solutions [20] and the film properties markedly depend on the moisture content [21]. MET comprises methylcellulose and three substitution types of hydroxypropylmethylcellulose (HPMC) each available in several grades (SM, SH and SE) with varying viscosities based on the type of esterification agent. The SM type is rich in methyl groups, SH type is rich in both hydroxypropyl and methyl groups while the SE type is rich in hydroxyethyl and methyl groups [22]. Some of the key properties of MET include solubility in cold water and forms transparent solutions; it forms reversible gels during heating due to its viscoelastic properties and the formed gel maintains its shape during heating [23,24].

In this study, we report on the stabilizing effects of βCD and γCD combined with l-arg for OME in polymer (MET) based mucoadhesive buccal films for potential delivery to paediatric patients. In addition, the films were functionally characterized for swelling, adhesiveness, dissolution of OME, its release kinetics and long-term stability over 28 days (four weeks). The originality of the research presented in this paper is the use of a composite stabilizing system combining CDs and l-arg within a polymer based mucoadhesive buccal formulation, which overcomes limitations of current oral gastrointestinal delivery.

## 2. Materials and Methods

The mucoadhesive polymer, Metolose (MET) was provided as a gift by Shin Etsu (Stevenage, Hertfordshire, UK). Polyethylene glycol (PEG 400), l-arginine (l-arg), gelatin and beta cyclodextrin (βCD) were purchased from Sigma-Aldrich (Gillingham, UK), ethanol, potassium dihydrogen phosphate and sodium hydroxide from Fisher Scientific (Leicester, UK) whilst omeprazole (OME) and gamma cyclodextrin (γCD) were purchased from TCI (Tokyo, Japan).

### 2.1. Formulation (Gel and Film) Development

Solvent cast films were formulated from ethanolic gels of the mucoadhesive polymer (MET) and loaded with drug (OME) as previously reported [17]. Briefly, the OME was dissolved in 20% *v*/*v* EtOH together with βCD or γCD at different ratios to form an OME solution as summarized in Table 1a. Subsequently, MET powder was slowly added to the vigorously stirred drug–CD solution at room temperature to obtain the drug loaded (DL) CD gels. The gels obtained gels were covered using parafilm, left to stand to allow the escape of air bubbles after which 20 g was poured into Petri dishes and left to dry in an oven set to a temperature of 40 °C [25]. Further, due to visually observed degradation of the drug even in the presence of either βCD or γCD alone in the ethanolic gel, l-arg was added in the CD containing gels as shown in Table 1b. During this step, l-arg (0.10% *w*/*w*) was incorporated into the gel whilst maintaining the concentration of the original OME (0.10% *w*/*w*), βCD (0.10% *w*/*w*), and γCD (0.10% *w*/*w*) constant [26].

### 2.2. Physico-Chemical Characterization

#### 2.2.1. Tensile Properties

A texture analyser (HD plus, Stable Micro System, Surrey, UK) equipped with a 5 kg load cell and texture exponent-32 software program was employed. Thickness and width of the films were measured and entered into the software program and used to calculate the tensile parameters. The films free from any physical defects, with the average thickness of (0.07 ± 0.01 mm) were selected for testing. The films which were cut in the shape of dumb-bell strips were attached to two tensile grips which were 30 mm apart and stretched at the speed of 1.0 mm/s till they broke. The tensile strength (representing film brittleness), elastic modulus (rigidity) and percentage elongation (flexibility and elasticity) were calculated using Equations (1)–(3). Each experiment was undertaken three times (*n* = 3) and average values calculated.

(1) $$Tensile\;strength=\;\frac{Force\;at\;failure}{cross-sectional\;area\;of\;the\;film}$$

(2) $$Percent\;elongation\;at\;break=\;\frac{Increase\;in\;length\;at\;break}{Initial\;film\;length}\;\times 100$$

(3) $$Young^{\prime }s\;modulus=\frac{slope\;of\;stres-strain\;curve}{Film\;thickness\;\times \;Cross\;head\;speed}$$

#### 2.2.2. Thermal Analysis

##### Hot Stage Microscopy (HSM)

These experiments were performed using a Mettler Toledo FP82HT (Greifensee, Switzerland) with a Nikon Microphot. Optimized MET DL films plasticized with 0.5% PEG 400, and containing OME:l-arg:βCD in ratio of 1:1:1 were placed on a glass slide, covered with a coverslip, and heated from ambient temperature to 200 °C at a rate of 10 °C/min. The changes in morphological behaviour with heating were collected in the form of a video recording with the help of a PixeLINK PL-A662 camera (PixeLINK, Ottawa, ON, Canada).

##### Differential Scanning Calorimetry (DSC)

DSC was performed for MET DL films analysed for HSM above and changes in their properties after the addition of PEG and OME within the films investigated. Small strips of each film (DL OME:l-arg:βCD 1:1:1), and starting materials (MET, OME and l-arg) weighing about 2.5 mg, were placed into hermetically sealed Tzero aluminium pans with a pin hole in the lid. The pans containing the samples were then heated in a Q2000 (TA Instruments, New Castle, DE, USA) calorimeter from a low temperature of 40 °C to 180 °C using a heating rate of 10 °C/min under constant flow of nitrogen (N_2_) (100 mL/min) to evaluate the glass transition, melting point, crystallization and a possible interactions between polymer and plasticizer [27].

##### Thermogravimetric Analysis (TGA)

TGA analyses were performed with the help of a Q5000 (TA Instruments, New Castle, DE, USA) thermogravimetric analyser to investigate the amounts of residual moisture within the films. About 1–2.5 mg (*n* = 3) of the optimized DL films (films prepared from ethanolic gels plasticized with 0.5% PEG 400, and containing OME:l-arg:βCD 1:1:1) were placed into hermetically sealed Tzero aluminium pans. The films were then heated from ambient temperature (20 °C) to 200 °C at a rate of 10 °C/min under nitrogen (N_2_) gas at a gas flow rate of 25 mL/min, to evaluate the residual moisture content of the starting materials (MET, OME, CD, l-arg) and DL films.

#### 2.2.3. Scanning Electron Microscopy (SEM)

The surface morphology, general uniformity and the presence of any cracks in the optimized MET DL films were investigated with SEM. Films were mounted onto Agar Scientific G301 aluminium pin-type stubs (12 mm diameter) with Agar Scientific G3347N double-sided adhesive carbon tapes and coated with chromium (Sputter Coater S150B, 15 nm thickness). The coated films were then evaluated using a Hitachi Triple detector CFE-SEM SU8030, (Hitachi High-Technologies, Tokyo, Japan) scanning electron microscope at an accelerating voltage of 2 kV [28,29].

#### 2.2.4. X-ray Diffraction (XRD)

XRD was used to analyse the physical form (crystalline or amorphous) of the optimized MET DL films. XRD diffractograms were obtained on a DIFFRAC plus instrument (Bruker, Coventry, UK) equipped with an XRD commander program. A Goebel mirror was used to produce a focused monochromatic CuKα_1&2_ primary beam (λ = 1.54184 Å) with exit slits of 0.6 mm and a Lynx eye detector and voltage and current settings set at 40 kV and 40 mA, respectively. The samples for analysis were prepared by cutting the films into 2 cm^2^ square strips to fit the square tiles of the holder. The films were subsequently mounted on the sample cell and then scanned between 2 theta of 0° to 70° with 0.1 s step size [30,31].

#### 2.2.5. Attenuated Total Reflectance Fourier Transform Infrared (ATR-FTIR) Spectroscopy

FTIR spectra were obtained using a Perkin Elmer spectrophotometer (Spectrum Two, Perkin Elmer, San Diego, CA, USA) equipped with a crystal diamond universal ATR sampling accessory (UATR). Prior to the start of each sample measurement, the ATR crystal was cleaned thoroughly using tissue paper soaked in ethanol. During the measurement, the films made intimate contact with the universal diamond ATR top-plate, enabled by a pressure clamp to hold samples in place throughout the analysis. For each sample, the spectra representing an average of 4 scans were recorded in the range of 4000–400 cm^−1^.

### 2.3. Functional Characterization

Functional characteristics were investigated for the selected optimized MET DL films (0.5% PEG 400, OME:l-arg:βCD 1:1:1).

#### 2.3.1. Swelling Index (Capacity)

The swelling capacities of the films were determined by incubating the samples in 0.01 M PBS solution at a pH of 6.8 ± 0.1 (to simulate salivary pH) and heated to a temperature of 37 ± 0.1 °C. The films were cut into 2 × 2 cm square strips and placed into Petri-dish containing 10 mL of the hydrating solution (PBS) and initially weighed. At predetermined time intervals (5 min), the PBS solution was completely removed using a syringe and weighed again. Before the films were weighed, excess PBS solution was gently off blotted using paper towels. After weighing, 10 mL of fresh PBS solution maintained at a temperature of 37 ± 0.1 °C was placed back in the Petri dish using a syringe. The experiments were performed in triplicate (*n* = 3) for each set of formulated samples and the percentage swelling index (swelling capacity) was calculated using Equation (4):

(4) $$Swelling\;Index\left(\%\right)=\frac{Ws-Wi}{Wi}\times 100$$

where *Ws* is the initial weight of the film before hydration and *Wi* is the initial weight of the film after hydration in the PBS solution.

#### 2.3.2. In Vitro Mucoadhesion

The in vitro mucoadhesion experiments were performed using the TA HD plus Texture Analyzer (Stable Micro Systems, Surrey, UK) fitted with a 5 kg load cell. The film was attached to an adhesive rig probe having a diameter of 75 mm using double sided adhesive tape. A model mucosal substrate was prepared by first dissolving 6.67 g of gelatin in 100 mL of warm deionized water and pouring 20 g of the warm solution into Petri dishes with diameter of 88 mm diameter and allowed to set as a solid gel. To allow proper simulation of the oral buccal mucosa and the surface of the set gelatin gel was equilibrated with 0.5 mL PBS (pH 6.8) to represent the buccal mucosa [32]. The film was attached to the texture analyser probe and placed in contact with the equilibrated gelatin gel for a period of 60 s to provide optimal contact and hydration. The following settings: pre-test speed 0.5 mm/s; test speed 0.5 mm/s; post-test speed 1.0 mm/s; applied force 1 N; trigger type auto; trigger force 0.05 N and return distance of 10.0 mm, in tension mode were used during the measurements and Texture Exponent 32 software was used to record and process the data. The peak adhesive force (PAF) required to completely detach the film from the gelatin surface was determined by the maximum force, area under the curve (AUC) representing the total work of adhesion (TWA) was estimated from the force-distance plot and the cohesiveness of the sample was determined by the distance travelled by the film before complete detachment.

#### 2.3.3. In Vitro Release of Omeprazole (OME) Using Franz-Type Diffusion Cell

Prior to investigating the drug release and dissolution profiles, drug assay and uniformity of OME within the film was determined. This was determined by first weighing the film accurately to a weight of 5 mg (*n* = 3) and hydrating in 8 mL of 0.01 M PBS solution at a pH of 6.8 and stirred at a temperature of 37 ± 0.5 °C until completely dissolved. The concentration of OME was analysed using HPLC (as described below).

For the in vitro drug dissolution studies, a Franz-type diffusion cell was used comprising donor compartment and receiver compartment. Five milligrams of the optimized MET DL film were placed in the donor compartment on stainless steel wire mesh (0.5 mm × 0.5 mm) which separated the donor and receiver compartments, with the mucoadhesive surface in contact with the wire mesh and facing the receiver compartment of the Franz diffusion cell [33]. The receiver chamber was filled with 8 mL of 0.01 M PBS pH 6.8, 37 °C and magnetically stirred at a speed of 250 rev/min. The chambers were held together with the help of a cell clamp and also sealed with parafilm to reduce evaporation. At predetermined time intervals, aliquots (1 mL) of the PBS medium was sampled and replaced with the same amount of fresh solution to keep the total volume constant over a 2 h period. The sampled dissolution medium was first filtered into an HPLC sample vial and analysed at 302 nm using HPLC (see Section 2.2.4). The concentration of OME released from the film was determined from the linearized calibration curve (R^2^ > 0.99) and percentage cumulative drug release profiles plotted. 

### 2.4. Drug Stability

Drug stability within the optimized films was determined by analysing the amount of drug present in the films after storing under two sets of conditions. Samples were placed in humidity controlled desiccators and placed in ovens at 40 °C representing accelerated conditions and the other at room temperature (ambient) over a period of 4 weeks. The MET DL film (OME:βCD:l-arg 1:1:1, PEG 400 (0.5% *w*/*w*) was placed in the desiccators and wrapped with aluminium foil due to its light sensitivity and to prevent moisture absorption by MET. For HPLC analysis, the stored samples were accurately weighed (5 mg) and hydrated in 0.01 M PBS solution (pH 6.8 ± 0.1 simulating salivary pH) in volumetric flasks (10 mL) and left to completely dissolve. Aliquots (1 mL) of the sample solution from each flask was removed and filtered into HPLC vials prior to analysis by HPLC. 

An Agilent 1200 HPLC machine equipped with auto sampler (Agilent Technology, Cheshire, UK) with Chemstation^®^ software program was used to analyse the samples. A Hypersil™ reversed phase ODS C18 HPLC column, with particle size of 5 µm and respective length and diameter of 250 mm × 4.6 mm (Thermo Scientific, Hampshire, UK), was used as the stationary phase. The mobile phase was prepared by mixing ammonium acetate and acetonitrile in the ratio of 60:40 *v*/*v*. The flow rate of the mobile phase was maintained at 2 mL/min and diode array UV detector wavelength for OME was set at 302 nm; with injection volume of 20 μL during each run.

## 3. Results and Discussion

### 3.1. Formulation Development and Optimization

The polymer (MET), which is a combination of hydroxypropylmethylcellulose and methyl cellulose, used in this study, was chosen because of its hydrophilic nature and well known swelling and mucoadhesive properties [34,35]. Stirring was applied during gel formulation to prevent formation of lumps which could occur through incomplete hydration, whilst heating the resulting mixture to a temperature of 40 °C reduced the viscosity of the final gels and helped air bubbles generated during stirring, to escape easily to the surface and subsequently released into the surrounding atmosphere. Furthermore, the reduced viscosity also allowed ease of pouring of the gels into the casting Petri dishes [36]. In addition, aqueous ethanolic solution (20% *v*/*v* of ethanol in water) was used as solvent because both OME and MET were more easily dissolved than water alone whilst also helping to reduce the drying time due to its volatility. 

#### Physical Evaluation of DL Films

One of the major challenges with OME is its physical and chemical instability, and therefore a key objective of this study was to stabilize the drug within the MET films using CDs either alone or in combination with l-arg. When OME is added to water, it dissolves quickly to produce a clear solution. OME dissolves very rapidly in the presence of water to yield a clear solution, and its stability after addition of MET and PEG, plays an important role in the overall stability of the final DL gel and subsequently the film formulation [37]. However, it was observed that the drug quickly showed signs of degradation within 20 min with the colour of the gel changing to red. This is because OME is only stable in alkaline solutions above pH 6.5.

**Figure 1:**
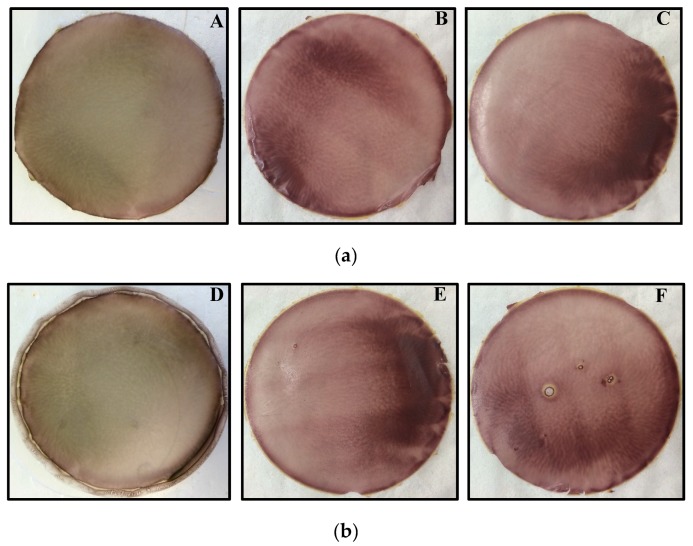
(**a**) Digital photographic images of OME and βCD loaded DL MET films in different OME:βCD ratios: (**A**) 1:1; (**B**) 1:2; and (**C**) 1:3 without l-arg, showing drug degradation indicated by brown coloration. (**b**) Digital photographic images of OME and **γ**CD DL MET films in different OME:**γ**CD ratios: (**D**) 1:1; (**E**) 1:2; and (**F**) 1:3 without l-arg showing drug degradation indicated by brown coloration.

**Figure 2:**
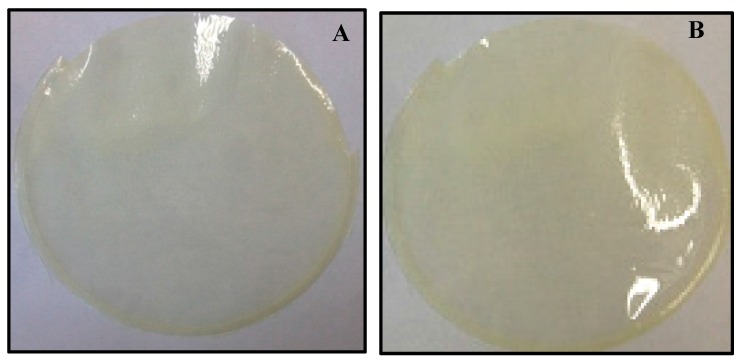
Photographs of OME and CD DL films: (**A**) βCD; and (**B**) **γ**CD with l-arg ratio (1:1:1) captured using a digital camera showing stabilization of OME in the presence of l-arg combined with CD.

The stabilization of OME was attempted by introducing two different types of CDs (β and γ) into the DL gel in three different ratios as shown in Table 1a,b. Although the OME loaded gels remained stable over a longer period (hours) in the presence of βCD and γCD, the colour of the gel eventually changed to brownish-red suggesting that both CDs on their own could not sustain the stability of the drug during gel and film formulation. The visual observations made for the final appearance of the different films were captured with a digital camera as shown in Figure 1. On the contrary, the OME-CD complex within the gels and the final dried films was stabilized in the presence of l-arg as shown in Figure 2, clearly depicting desirable and optimum properties of homogeneity, transparency and uniform drug distribution. This is because OME is unstable at low pH but this improves at higher pH and the presence of l-arg provides a neutral to weakly alkaline environment which improved the stability.

The molecular mechanism of how l-arg and OME interact with βCD by the formation of inclusion complexes, has been reported in the literature based on the work by Figuerias and co-workers [38]. They showed that with the presence of l-arg, the difference observed in the mean distances between the “e” and “f” atoms of OME to the centre of mass of CD’s O4 atoms, which was considered as a reference to the centre of the CD inner cavity of the two complexes, was not significant [38]. They further suggested that for OME:l-arg combined in equal ratios, the H atom of the l-arg was nearer in proximity to the nitrogen atom of OME and noted that the distance between the H (l-arg) and the N (OME) was relatively small, and explained that this increased the probability of hydrogen bonds forming between the two compounds. The visual characterization for the physical appearance of the films based on the most ideal characteristics (transparency, ease of peeling and flexibility) are summarized in Table 2. In summary, based on the visual evaluation, it was determined that DL MET films combining either l-arg and βCD or l-arg and **γ**CD in the ratio of OME:CD:l-arg 1:1:1 were most optimum and were therefore selected for further analytical and functional characterization studies. 

### 3.2. Physico-Chemical Characterization

#### 3.2.1. Texture Analysis (TA)

**Figure 3:**
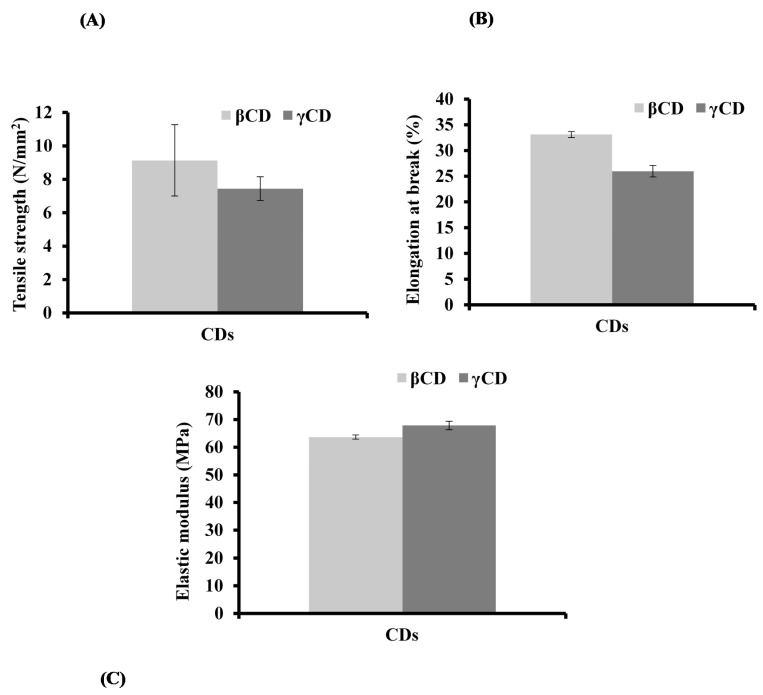
Tensile properties: (**A**) tensile strength; (**B**) elongation at break; and (**C**) elastic modulus of DL MET films prepared from ethanolic gels containing (0.5% *w*/*w* PEG 400), OME:βCD:l-arg (1:1:1) and OME:γCD:l-arg (1:1:1) (mean ± SD, (*n* = 3)).

Texture analysis was used to characterize the tensile behaviour of the films by measuring tensile strength, elastic modulus and per cent elongation at break of the DL films. The tensile properties were used to evaluate the effects of OME, βCD, γCD and l-arg on the behaviour of the plasticized MET films and the results used to further select the most appropriate formulations for further analysis. The effects of CD on the tensile strength values of the films are shown in Figure 3 and showed significant differences (*p* < 0.05) in the tensile strength (brittleness).

It has been reported that, ideally, the mean per cent elongation at break for thin films should fall somewhere between values of 30 to 60% [36] which is indicative of an appropriate balance between flexibility and elasticity. The βCD loaded film satisfied this required criterion, however, γCD loaded films gave a low per cent elongation at break as shown in Figure 3. Based on the previously identified ideal characteristics for a good film in terms of flexibility, uniformity and transparency, the formulations prepared from ethanolic gels containing 1:1:1 ratio of OME:βCD:l-arg and plasticised with 0.5% *w*/*w* PEG400 was confirmed to be the most appropriate for further investigations.

#### 3.2.2. Thermal Analysis

##### Hot Stage Microscopy (HSM)

**Figure 4:**
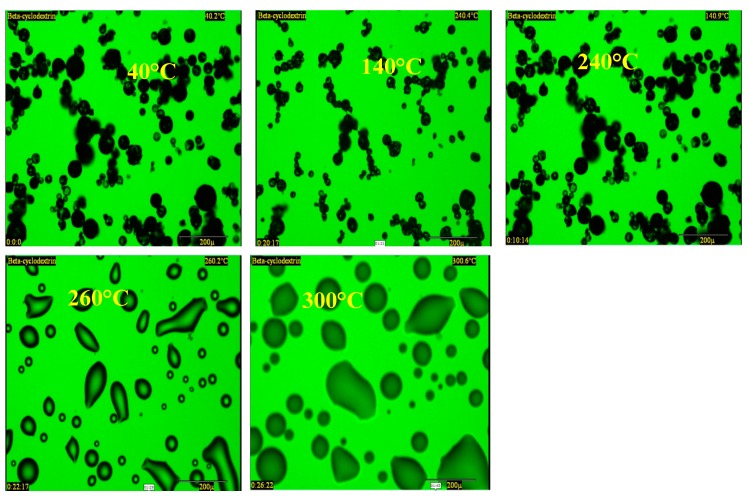
Hot stage microscopy (HSM) results showing selected optimized DL MET film containing βCD.

The HSM results were used to aid the development of appropriate heating cycles during the TGA and DSC analyses and helped to determine the maximum temperature to which the samples could be heated. For the selected βCD containing films, the results showed that with increase in temperature, the surface of the film changed from rough to clear as a result of water evaporation from the film matrix as shown below in Figure 4. It has been reported in the literature [39] that βCD undergoes a melt transition between 290 °C and 300 °C. However, Figure 4 shows melting after 260 °C, followed immediately by decomposition.

##### Differential Scanning Calorimetry (DSC)

**Figure 5:**
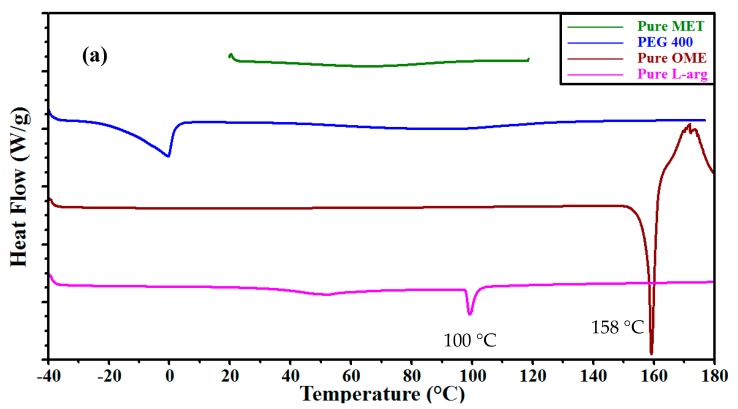

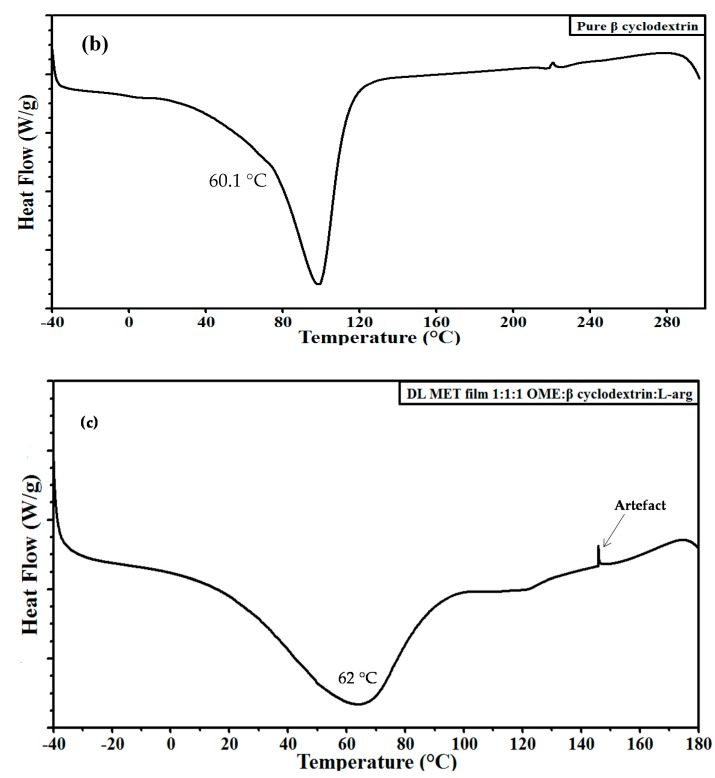
Differential scanning calorimetry (DSC) thermograms for: (**a**) the pure metolose (MET), pure omeprazole (OME), pure l-arginine (l-arg) and polyethylene glycol (PEG 400); (**b**) pure beta cyclodextrin (βCD); and (**c**) plasticized drug loaded (DL) MET films from gels loaded with 0.5% *w*/*w* PEG 400 and containing OME:βCD:l-arg (1:1:1).

DSC was employed to investigate the interactions between the components (MET, PEG 400, l-arg and model drug (OME)) of the formulation within the film matrix as shown in Figure 5a. The polymer (MET) showed a broad endothermic peak between 60–80 °C, caused by evaporation of water and no definite melt or glass transition peak, whilst pure OME showed a melting peak at 158 °C and l-arg at 100 °C, with pure βCD showing broad endothermic peak at 60.1 °C attributed to water loss with no definite melt or glass transition peaks (Figure 5b). The thermograms (Figure 5c) of plasticized MET DL films loaded with OME:βCD:l-arg in equal proportions, exhibited a broad endothermic transition with peak temperature of 62 °C and no melt peaks for OME and l-arg, suggesting films were amorphous. However, SEM and XRD analyses were further undertaken to confirm this definitively.

##### Thermogravimetric Analysis (TGA)

TGA was employed to quantify the residual water content as a percentage of the total film weight. The water content for βCD after heating from ambient temperature (20 °C) to 100 °C was 13.09% whilst the MET DL films containing OME:βCD:l-arg (1:1:1) had a residual water content of 4.04%. The residual water was significantly lower for films compared to the pure βCD which is due to EtOH in the gels which increased the rate of water evaporation during drying to obtain the films coupled with the oven drying temperature of 40 °C. Such low residual moisture is expected to contribute towards the slower rate of OME degradation by hydrolysis, which is important given the known poor stability of OME.

#### 3.2.3. Scanning Electron Microscopy (SEM)

**Figure 6:**
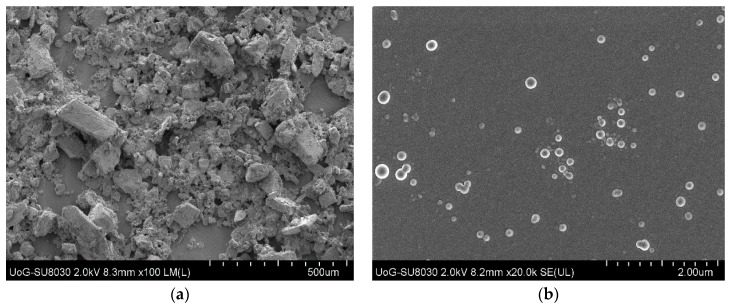
Scanning electron microscopy (SEM) micrograph of: (**a**) pure beta cyclodextrin (βCD); and (**b**) plasticized drug loaded (DL) metolose (MET) films cast from ethanolic (20% *v*/*v* EtOH) gels containing 0.5% *w*/*w* PEG 400 and OME:βCD:l-arg (1:1:1).

The morphology and topographic appearance of DL MET films containing drug (OME) and stabilizers (l-arg and βCD) were analysed with the help of SEM, as shown in Figure 6. The results show that pure βCD appeared as irregular particles without any well-defined shapes.

The topography of the plasticized films containing OME:βCD:l-arg (1:1:1) showed continuous sheets with relatively smooth and homogeneous surfaces and no pores observed. This suggests that all formulation constituents were uniformly mixed during gel and film formation. However, small structures with circular shapes can be seen on the film surface and could be attributed to excess OME or βCD precipitating out due to the rapid evaporation of ethanol and water during drying and film formation, but this requires further evaluation.

#### 3.2.4. X-ray Diffraction (XRD)

**Figure 7:**
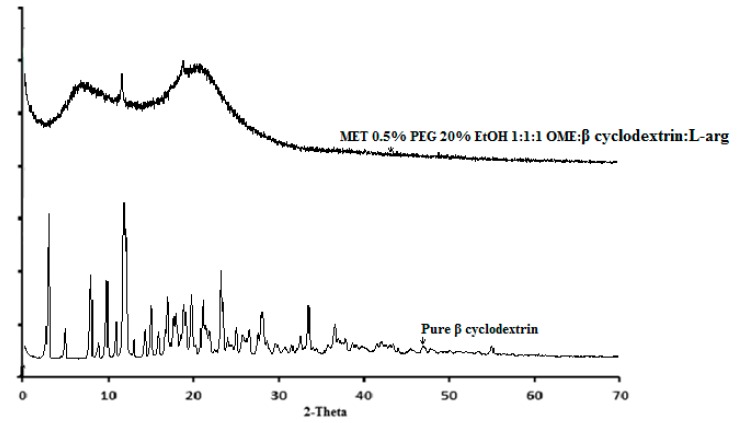
XRD diffractograms for pure beta cyclodextrin (βCD) and drug loaded (DL) metolose (MET) films cast from ethanolic (20% *v*/*v* EtOH) polymeric gels containing 0.5% *w*/*w* polyethylene glycol (PEG 400) and OME:βCD:l-arg (1:1:1).

XRD analysis was performed to investigate the estimated crystalline–amorphous ratio and confirm the physical form of the various components of the films. The XRD diffractograms of the pure βCD and DL MET films cast from ethanolic gels (20% *v*/*v* EtOH) gels containing OME:βCD:l-arg (1:1:1) with 0.5% *w*/*w* PEG 400 are shown in Figure 7. 

The results show that pure βCD was crystalline and confirmed the DSC results. In addition, small additional peaks corresponding to pure OME and βCD were observed, which indicate small levels of crystallinity within the plasticized film, however, overall, the films were generally amorphous. The crystalline:amorphous ratio was estimated at 2% which suggests there could be small amounts of free OME and/or βCD present in these films and could be attributed to the small amounts of recrystallized structures present on the film surface according to the SEM images. 

#### 3.2.5. Fourier Transform Infrared (FTIR) Spectroscopy

**Figure 8:**
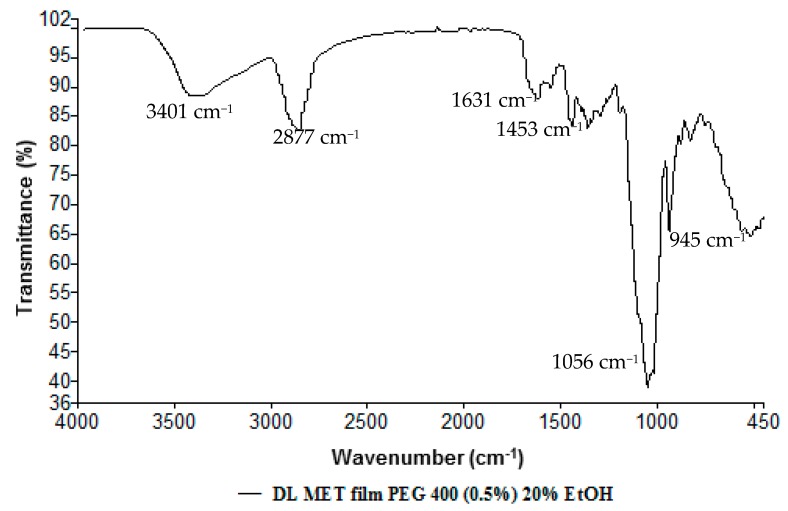
FTIR spectra of drug loaded (DL) metolose (MET) films obtained from ethanolic gels comprising 0.5% *w*/*w* polyethylene glycol (PEG 400) and OME:βCD:l-arg (1:1:1).

FTIR analysis was employed to investigate molecular changes in the drug due to any interactions with the other additives present within the film matrix. The FTIR absorption bands of βCD are summarized in Table 3 whilst those for plasticized DL MET are shown in Figure 8. The FTIR spectra of DL MET films, showed the characteristic absorption bands of OME decreased in intensity which could be due to dilution occurring from addition of βCD. There were no new bands observed in the spectrum, which suggests that no new entities were generated from interaction between the film components, as shown in Figure 8. 

### 3.3. Functional Characterization

#### 3.3.1. Hydration (Swelling) Capacities 

Plasticised DL MET films containing drug (OME) and stabilizer (l-arg) (ratio: 1:1), without βCD, showed swelling index of 2630% in 20 min. After 20 min, the swelling remained constant or decreased (data not shown) due to a loss of structural integrity. On the other hand, DL film containing βCD in addition to l-arg showed swelling index value of 1197% after 20 min, which was significantly lower (*p* < 0.05) compared to the above DL film with no βCD. This can be attributed to the interaction between MET and βCD complex within the film matrix and therefore competing with water molecules for bonding interactions with the polymer chains with a consequent decrease in hydration rate and eventual swelling capacity. This is an interesting observation because it is generally known that CDs are highly hydrophilic due to a high number of available OH groups.

#### 3.3.2. Mucoadhesion

**Figure 9:**
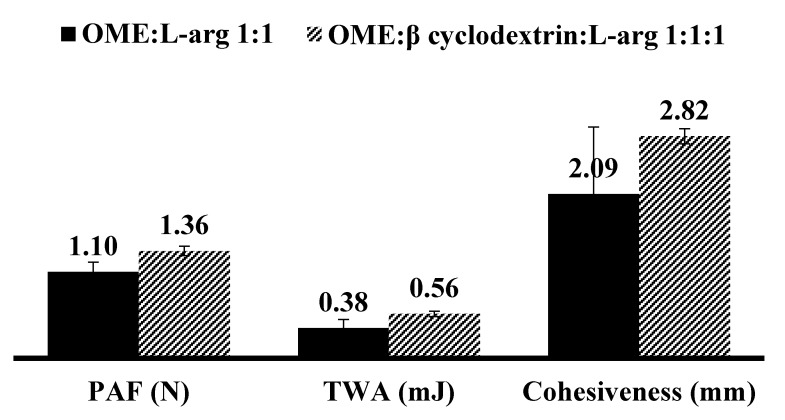
In vitro mucoadhesion profiles (peak adhesive force—PAF, total work of adhesion—TWA and cohesiveness) of plasticized drug loaded (DL) metolose (MET) film cast from ethanolic (20% *v*/*v* EtOH) gels containing OME:βCD:l-arg (1:1:1) using mucosal substrate equilibrated with phosphate buffered saline (PBS) (pH 6.8) of (mean ± SD, (*n* = 3)).

Figure 9 shows mucoadhesion data for DL MET film with and without βCD and the results show statistically significant differences for PAF (stickiness) (*p* = 0.0284) and TWA (*p* = 0.0522) whilst differences between their cohesiveness values were not significant (*p* = 0.2136). This could be attributed to strong interaction (hydrogen bonding) between the polymer (MET) and the βCD both of which contain large numbers of OH side groups, therefore reducing interaction with the model mucosa surface. 

#### 3.3.3. In Vitro Release of OME Using Franz-type Diffusion Cell

**Figure 10:**
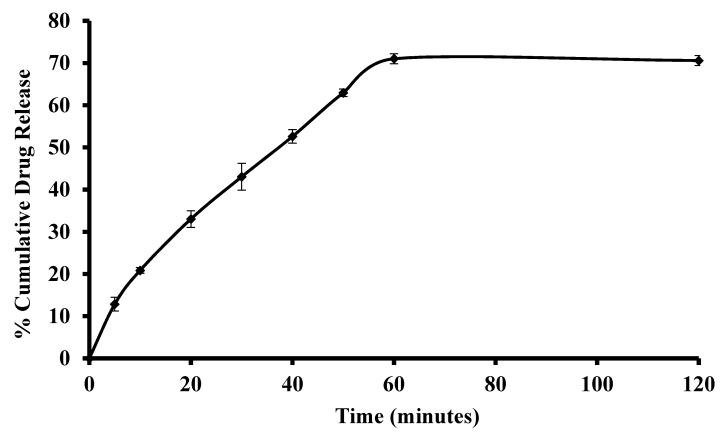
Drug dissolution profile of drug loaded (DL) metolose (MET) films prepared from ethanolic (20% *v*/*v* EtOH) gel containing 0.5% *w*/*w* PEG 400 and OME:βCD:l-arg 1:1:1 ratio in PBS at pH 6.8 (mean ± SD,(*n* = 3)).

The release of drugs from matrix delivery systems such as films, can be controlled by diffusion from the swollen matrix, by erosion of the polymer matrix or by a combination of both drug diffusion and subsequent erosion of the matrix [40]. The dissolution profiles of the optimized OME loaded films were observed over a period of two hours because, after 60 min, the percentage of OME release remained constant. Furthermore, 2 h is the most realistic time frame for holding a formulation on the buccal mucosa surface before complete hydration or possible dislodging due to chewing or tongue movements [41]. Figure 10 shows the dissolution profile of the selected optimized DL MET film containing OME:βCD:l-arg (1:1:1) in PBS (pH 6.8). The profile shows an initial linear release phase for the first 60 min with the release reaching a maximum of 70% which did not change beyond 60 min. 

The mechanism of OME release was investigated by fitting the drug release data to various kinetic models: zero order, first order, Higuchi and Korsmeyer–Peppas equations (coefficient, R^2^ values) [42]. The release kinetics of OME in PBS (pH 6.8) showed that the drug release mechanism followed Korsmeyer–Peppas model as the R^2^ value (0.9996) was the highest compared to other models. Further, evaluation of the release exponents (*n*) provides additional information regarding the specific molecular mechanism which controls drug release. OME release data from DL MET film containing βCD gave an *n* value of 0.6 which was greater than 0.45, which indicates that the drug release followed Fickian diffusion mechanism. This suggests that the OME was released through the hydrated (swollen) polymer via diffusion combined with erosion controlled drug release.

### 3.4. Drug Stability

**Figure 11:**
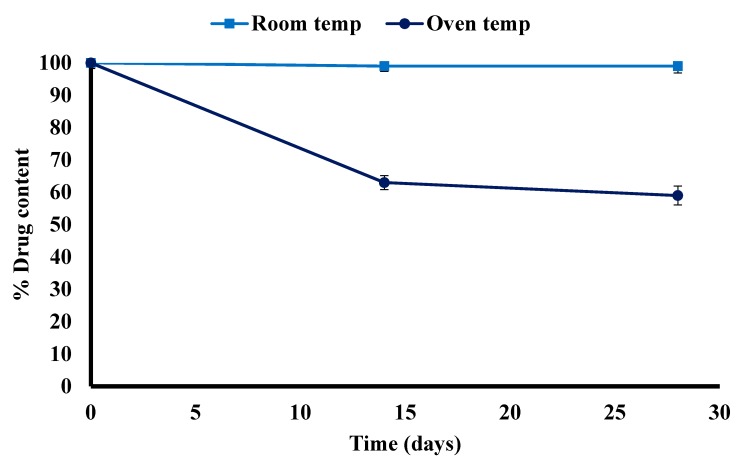
Plot showing the percentage of drug remaining within the film during storage at room temperature and oven temperature (40 °C) up to one month (mean ± SD, (*n* = 3)).

The chemical stability of OME within the optimized MET DL film (OME:βCD:l-arg 1:1:1, PEG 400 (0.5% *w*/*w*), 20% *v*/*v* EtOH) was determined by way of a short-term stability study following ICH guidelines. The films were placed in desiccators and wrapped with aluminium foil due to OME’s known light sensitivity. The films were also wrapped in paraffin film to prevent moisture absorption by MET and exposed to accelerated (40 ± 0.5 °C) and room temperature (ambient ± 0.5 °C) conditions (ICH guidelines) for a period of 28 days (4 weeks). The results depicted in Figure 11 show that there was a statistically significant difference (*p* < 0.05) between the per cent drug remaining within the film kept in the oven at 40 ± 0.5 °C compared to the one stored under ambient conditions with the latter remaining stable during the storage period. In the first 14 days, the drug content of OME in films kept at 40 ± 0.5 °C was 63% compared to 99% at room temperature and after 28 days the content of OME was 59% at 40 ± 0.5 °C and 99% at room temperature. These observations suggest that, when βCD and l-arg are introduced into the film, it maintains the stability of OME at room temperature (ambient ± 0.5 °C) conditions but not under accelerated conditions of 40 ± 0.5 °C. The results give an indication of the appropriate storage conditions for the formulated CD loaded films to maintain the stability of OME possibly prolonging shelf life.

## 4. Conclusions

This study has shown the successful incorporation of both βCD and l-arg into OME loaded mucoadhesive buccal films prepared from MET for potential treatment of GERD in children. From the results obtained, it was shown that, when βCD was incorporated into the original optimized DL MET film, it did not significantly affect the DSC and FTIR profiles and drug release characteristics. However, there were changes observed in TGA, SEM, XRD, swelling and mucoadhesion properties. Furthermore, the OME present in βCD loaded mucoadhesive films remained stable at room temperature over a 28-day period. This study has also shown that, although βCD can stabilize OME, the short to long term stability of OME by βCD is also dependent on l-arg, which is known to increase pH to improve the overall stability of the drug. Finally, OME was most stable at room temperature over the 28 days compared to accelerated temperature of 40 °C, suggesting that the ideal environment to store the βCD/l-arg stabilized mucoadhesive films will be under ambient temperature and humidity conditions.

## Figures and Tables

**Table 1:** (**a**) Composition of omeprazole (OME) loaded metolose (MET) ethanolic gels containing varying concentrations of amounts of beta cyclodextrin (βCD) and gamma cyclodextrin (γCD), polyethylene glycol 400 (PEG 400) at a concentration of 0.50% *w*/*w*; and (**b**) optimized gels containing 0.50% *w*/*w* PEG 400, βCD and γCD as well as l-arginine (l-arg) in ratio of OME:CD:l-arg 1:1:1.

Gel Formulation Composition	Concentration (% *w*/*w*)			
	(a)			(b)
MET	1.0			1.0
OME	0.1			0.1
PEG 400	0.5			0.5
βCD	0.1	0.2	0.3	0.1
γCD	0.1	0.2	0.3	0.1
l-arg	0			0.1

**Table 2:** Ideal characteristics of optimized drug loaded (DL) films of βCD and γCD.

**Polymer**	**Solvent**	**PEG (% *w*/*w*)**	**OME (g)**	**βCD (g)**	**l-arg (g)**	**Ease of Peeling**	**Film Characteristics**
MET	20% EtOH	0.5	0.1	0.1	0.1	YES	Transparent/flexible
**Polymer**	**Solvent**	**PEG (% *w*/*w*)**	**OME (g)**	**γCD (g)**	**l-arg (g)**	**Ease of Peeling**	**Film Characteristics**
MET	20% EtOH	0.5	0.1	0.1	0.1	YES	Transparent/flexible

**Table 3:** The observed characteristic FTIR bands for pure cyclodextrin (CD).

Pure Materials	Absorption Bands (cm^−1^)	Bands Assignment
βCD	998	C–H bending
	1077	–S=O stretching
	1152	C=O stretching
	1415	C–H stretching
	1644	–C=C stretching
	2925	C–H stretching
	3295	N–H stretching
